# Review on Mechanical Support and Cell-Based Therapies for the Prevention and Recovery of the Failed Fontan-Kreutzer Circulation

**DOI:** 10.3389/fped.2020.627660

**Published:** 2021-01-26

**Authors:** Margaret R. Ferrari, Michael V. Di Maria, Jeffrey G. Jacot

**Affiliations:** ^1^Department of Bioengineering, University of Colorado Anschutz Medical Campus, Aurora, CO, United States; ^2^Division of Cardiology, Heart Institute, Children's Hospital Colorado, University of Colorado Anschutz Medical Campus, Aurora, CO, United States; ^3^Department of Pediatrics, Children's Hospital Colorado, University of Colorado Anschutz Medical Campus, Aurora, CO, United States

**Keywords:** mechanical assist devices, stem cell therapies, hypoplastic left heart syndrome, Fontan failure, right ventricular dysfunction, cardiac tissue engineering, single ventricle disease

## Abstract

Though the current staged surgical strategy for palliation of single ventricle heart disease, culminating in a Fontan circulation, has increased short-term survival, mounting evidence has shown that the single ventricle, especially a morphologic right ventricle (RV), is inadequate for long-term circulatory support. In addition to high rates of ventricular failure, high central venous pressures (CVP) lead to liver fibrosis or cirrhosis, lymphatic dysfunction, kidney failure, and other comorbidities. In this review, we discuss the complications seen with Fontan physiology, including causes of ventricular and multi-organ failure. We then evaluate the clinical use, results, and limitations of long-term mechanical assist devices intended to reduce RV work and high CVP, as well as biological therapies for failed Fontan circulations. Finally, we discuss experimental tissue engineering solutions designed to prevent Fontan circulation failure and evaluate knowledge gaps and needed technology development to realize a more robust single ventricle therapy.

## Introduction

Single ventricle heart disease (SVD) is a complex and severe congenital heart defect (CHD) that is characterized by a single pumping chamber and an inability to septate the heart surgically into dedicated pulmonary and systemic circulations. SVD represents roughly 3% of all CHD cases and may affect several structural components of the heart including the valves, ventricles, aorta, and septa (1). Treatment for SVD has changed drastically over the past several decades. This once fatal disease now has surgical treatment options that carry patients into early adulthood. However, the standard of care approach does not adequately support patients long-term (2) and many researchers are investigating novel bioengineered solutions to treat SVD. This review will detail the significant advances in bioengineered solutions to the challenges facing patients after the Fontan-Kreutzer surgery.

### Standard of Care

**Figure 1:**
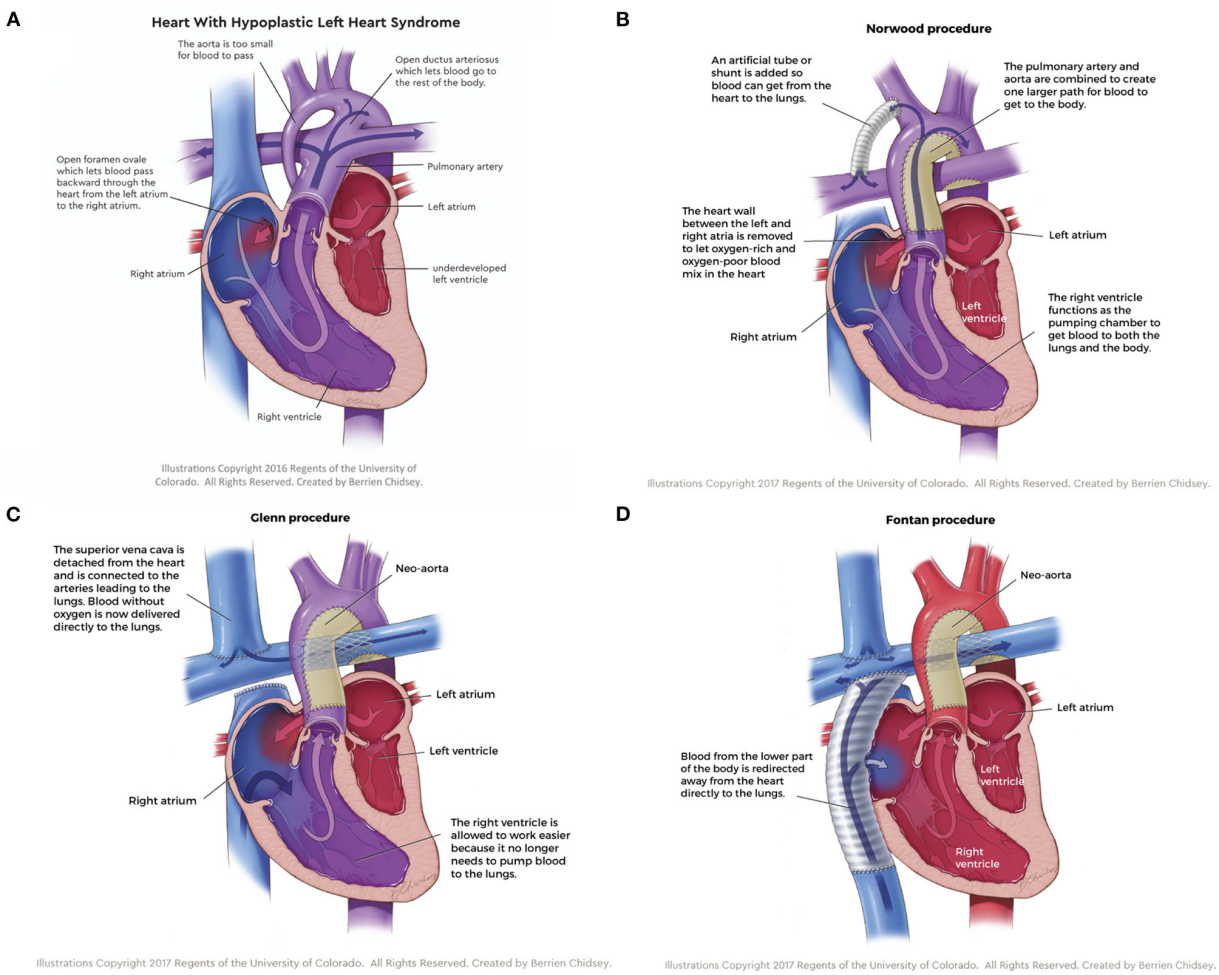
**(A)** Hypoplastic left heart syndrome, the most common type of single ventricle disease, is characterized by an underdeveloped left side of the heart. **(B)** The stage I Norwood procedure establishes an unobstructed pathway from the ventricle to the body and diverts a pressure-limited supply of blood to the lungs. **(C)** The stage II Glenn procedure directs all superior systemic venous flow directly to the pulmonary artery, which reduces the volume load on the single ventricle, thereby decreasing wall stress and ventricle wall hypertrophy. **(D)** The stage III Fontan-Kreutzer procedure places the pulmonary and systemic circulation in series, introducing passive flow into the pulmonary circuit while the functional right ventricle pumps oxygenated blood to the systemic circulation.

For the purposes of this review, the focus will be on hypoplastic left heart syndrome (HLHS), the most common type of SVD where the left heart structures are underdeveloped (Figure 1A); however, many of the therapies reviewed are applicable to other SVD physiologies. A three-stage palliative surgical strategy is the most common treatment option for HLHS patients. The first surgery, the stage I Norwood procedure, occurs within a few days of birth. In the Norwood operation, surgeons establish an unobstructed pathway from the ventricle to the body and divert a pressure-limited supply of blood to the lungs (Figure 1B). To this end, a “neo-aorta” is constructed using a pulmonary homograft, xenogeneic pericardium, or a synthetic graft and connected to the right ventricle (RV), allowing the functional pumping chamber to supply the systemic circulation (3). Additionally, an atrial septal defect is created (or enlarged) to ensure oxygenated blood returning to the left atrium can access the single ventricle. Pulmonary flow is directed to the lungs using a Blalock-Taussig (BT) shunt; typically, a 3.5-millimeter conduit is anastomosed between the right pulmonary artery (PA) and the right subclavian artery (3). Alternatively, surgeons can place a Sano shunt, a conduit between the RV and PA (4). While not in common use in most surgical centers in the US, the hybrid procedure (Patent ductus arteriosus stenting and bilateral PA banding) can also be implemented in place of the Norwood operation as an initial palliative procedure. The second palliative procedure, the Bidirectional Glenn, occurs 4–6 months after the Norwood operation and directs all superior systemic venous flow into the pulmonary artery (Figure 1C) (5). The BT shunt is removed, and the superior vena cava (SVC) is anastomosed to the proximal right pulmonary artery. This procedure reduces the volume load on the single ventricle, thereby decreasing wall stress and ventricle wall hypertrophy (6). The third and final surgery, the Fontan-Kreutzer procedure, is implemented between 2 and 4 years of age, placing the pulmonary, and systemic circulation in series (Figure 1D). A synthetic conduit is commonly used to connect the inferior vena cava (IVC) directly to the PA (7). In conclusion, staged palliation introduces passive flow into the pulmonary circuit via an IVC and SVC anastomotic connection directly to the pulmonary artery, and the functional right ventricle pumps oxygenated blood to the systemic circulation. This passive pulmonary flow is accompanied by obligate elevation in central venous pressure (CVP), leading to immediate post-operative and also life-long obstacles (8).

### Post-surgical Complications

While the series of palliative procedures culminating in the Fontan-Kreutzer operation addresses the issues of obstructed systemic blood flow and cyanosis, it introduces a host of other complications. Heart failure and circulatory dysfunction remain difficult to characterize in this heterogenous population and deviate from the classical biventricular heart failure phenotype. Fontan failure has several different presentations, ranging from a phenotype similar to that of traditional “right heart failure” to inexorable lymphatic leak (9). As a result of the series circulations and the lack of a sub-pulmonary ventricle, CVP is significantly elevated. This is the primary driver of multiple different presentations of “Fontan failure,” including protein losing enteropathy (PLE), plastic bronchitis (PB), and liver fibrosis and cirrhosis (10–12).

The thoracic duct, which drains mesenteric and thoracic lymphatics, inserts into the innominate vein, which has a pressure of 12–15 mmHg after the Fontan-Kreutzer operation (normal CVP is 3–5 mmHg). As a result, there is a decrease in the pressure gradient driving lymphatic flow, which impairs lymphatic drainage with increases the risk of lymphatic dysfunction and failure. Experts believe PLE is due to retrograde drainage of lymphatic fluid into the intestinal tract after a direct connection between the lymphatics and the gut lumen has been established, for example, following an acute gastroenteritis (13). PLE leads to complications such as bone density reduction, coagulation irregularities, abdominal swelling, immune dysfunction, and abnormal linear growth. When lymphatic leak occurs into the hollow organs of the chest, lymphatic fluid drains via collaterals into the airways and manifests as plastic bronchitis. A thick, rubbery cast forms in the airway and may block air flow, leading to hypoxemia and in the worst case possible suffocation if not removed or expectorated (11). The presence of PB and PLE have been associated with up to a 16-fold increase in the risk of mortality, Fontan takedown, or heart transplant (14, 15).

Another common issue in this patient population is acquired Fontan-associated liver disease (FALD). Hepatic arterial flow is altered in the Fontan circulation and studies have indicated that a combination of abnormal perfusion of the liver combined with hepatic congestion can lead to hepatic fibrosis, which can lead to hepatic cirrhosis or even hepatocellular carcinoma with prolonged fibrotic scarring (16, 17). While the implications of FALD remain incompletely understood, there are important considerations related to heart transplantation as a therapeutic option in end-stage Fontan failure (18).

Studies have indicated that the number of Fontan-associated comorbidities are directly related to age and result in increased risk of death, length of hospital stay, and cost of healthcare (14, 19, 20). Thus, as patients age, the number of post-Fontan complications increases as well as their risk of death. A recent study suggests over half of Fontan patients present with heart failure, require heart transplantation, or die within a 28 years' time period (14). This data indicates that the standard of care falls short in supporting this population long-term and incentivizes the research community to develop novel treatment strategies for infants and adults with SVD.

### Options for Patients With Fontan Failure

Once circulatory failure is apparent, heart transplantation is the only defined option for patients after clinical decompensation. Outcomes of heart transplant in this population vary widely between centers and single ventricle anatomy (21–23). Graft failure, infection, and operative complications following heart transplant are more common in the Fontan population compared to other CHD conditions (22–25). In fact, one report suggested patients that previously received a Fontan-Kreutzer operation were 8.6 times more likely to die after cardiac transplantation than patients with other CHD anomalies (25). While overall survival outcomes in pediatric heart transplantation have increased over the past decades, donor hearts are still scarce, limiting the availability of heart transplantation for the failed Fontan population. In fact, one study found that 21.1% of pediatric patients listed in the high risk category died while waiting for a suitable donor (26). However, several reports suggest temporary mechanical circulatory support can sustain a patient for a short time while waiting for cardiac transplantation (27–31). This option is often referred to as a “bridge-to-transplantation” and while it has shown promising short-term outcomes, many SVD patients still die waiting for a donor heart (24).

Advancements in research have combined scaffold and stem cell technologies for prevention and reversal of Fontan failure. These technologies will be reviewed here, in addition to analyses of other bioengineered solutions. Further, this review will compile and detail design considerations for a preventative, tissue engineered solution for the SVD population. This is the first review, to our knowledge, that summarizes both preemptive and reactionary bioengineered solutions for the Fontan patient.

## Mechanical Solutions for Patients With Fontan Failure

As circulatory mechanical support device (MSD) technology develops, these devices are increasingly used in the single ventricle population. As previously mentioned, in the short-term, these devices are used for “bridge-to-transplantation” or emergency situations. Extracorporeal membrane oxygenation (ECMO) is used to support pediatric patients with cardiopulmonary failure and represents the first MSD, albeit with an oxygen delivery component, used in patients with SVD. However, reports suggest patients with single ventricle physiology on ECMO are less likely to survive compared to children with biventricular CHDs (67% in hospital mortality vs. 56%) (32). Similarly, an analysis of data acquired by the Extracorporeal Life Support Organization Registry reported neonatal SVD patients have a 65% mortality rate on ECMO, nearly 10% higher than in patients with biventricular CHDs (33). Additionally, the risk of death for patients with single ventricle physiology on ECMO increases drastically with time, specifically after 6 days (32–35). This established outcome data has led to a surge in the use of implantable MSDs, rather than extracorporeal, for indefinite support of SVD patients. This section will briefly identify the most common long-term MSDs that have been implanted in patients with a failing Fontan circulation. Reviews devoted to MSDs for patients with single ventricle physiology have been published and should be referenced for a detailed landscape of all mechanical assist options (36–39).

### Mechanical Support Devices Implanted in Patients With Fontan Failure

The first MSD developed for long-term support of pediatric heart failure patients has appeared within the last several decades—The Berlin Heart (BH). This device is a pneumatically driven extracorporeal ventricular assist device (VAD) that was approved by the Food and Drug Administration (FDA) under a Humanitarian Device Exemption in 2011 (HDE #H100004). This device has the ability to support patients with Fontan circulation until cardiac transplantation (27, 29, 31, 32, 40–50). Details regarding each implantation can be found in Table 1. The BH has supported adults (older than 18 years) with a failed Fontan circulation for up to 493 days (55) and pediatric patients with a failed Fontan for up to 229 days (51). The youngest pediatric patient to receive a BH with successful bridge to transplantation was 15 months old and supported for 7 weeks (50). Studies report the BH can also lower CVP levels in a pediatric patient by 4 mmHg (27) and an adult patient by 22 mmHg (31). These are promising results for those with high CVP levels, the primary clinical driver of failed Fontan circulation.

**Table 1:** Comprehensive list of case studies and retrospective studies where long term, mechanical ventricular assist devices were implanted in patients, adult and pediatric, with a failed Fontan circulation.

**References**	**Long-term cardiac support device**	**Total patients (peds/adult)**	**SVD patients**	**Outcome measure**	**Complications**	**Survived**	**Died**
Weinstein et al. (51, 52)	Berlin Heart EXCOR	281 peds	26°	Duration of support: ≤ 229 days Bridge to transplant: 10 patients	Stroke Multiorgan failure Respiratory failure Bleeding	11°	15
Napoleone et al. (42)	Berlin Heart EXCOR	1 (5 yr-o)	1	Duration of support: 3 months Bridged to transplant: yes	None reported	1	0
VanderPluym et al. (44)	Berlin heart EXCOR	1 (2.8 yr-o)	1	Duration of support: 44 days Bridged to transplant: No	Thrombus Diuresis Respiratory failure Elevated CVP Reversal of blood flow Mesenteric ischemia	0	1
Calvaruso et al. (29)	Berlin heart EXCOR	1 (10 yr-o)	1	Duration of support: 10 days Bridged to transplant: yes	Insufficient ventricular filling Pacemaker implantation	1	0
VanderPluym et al. (43)	Berlin heart EXCOR	1 (3 yr-o)	1	Duration of support: 174 days Bridged to transplant: Yes	Subdural hemorrhage Decompressive craniotomy Fibrin collection on valves Pump changes	1	0
Hoganson et al. (27)	Berlin Heart EXCOR	1 (4 yr-o)	1	Duration of support: 26 days Bridged to transplant: Yes O_2_ Saturations: 96-100% CVP: 4 mmHg	Pump thrombi	1	0
Valeske et al. (53)	Berlin Heart EXCOR	1 (19 yr-o)	1	Duration of support: 23 days Bridged to transplant: yes	Hemolysis Blood transfusion Increased CVP Ascites Polyneuropathy Sepsis	1	0
Seitz et al. (45)	Berlin Heart EXCOR (post HT)	1 (2.5 yr-o)	1	Duration of support: 20 weeks Recovered RV function: yes Weaned off device: yes	Pump changes PLE relapse Infection	1	0
Petre et al. (31)	Berlin Heart	1 (27 yr-o)	1	Duration of support: 13 months ΔCVP: 22 mmHg Bridged to transplant: yes	None reported	1	0
Nathan et al. (46)	Berlin Heart VAD	1 (4 yr-o)	1	Duration of support: 28 days Bridge to transplant: yes	Multiorgan failure Primary graft failure	0	1
Pearce et al. (50)	Berlin Heart VAD	1 (15 mths)	1	Duration of support: 7 weeks Bridged to transplant: yes	None reported	1	0
Cardarelli et al. (48)	Berlin Heart VAD	1 (1.5 yr-o)	1	Duration of support: 179 days	Thrombus Stroke Ascites Tracheostomy	0	1
Halaweish et al. (47)	Berlin Heart VAD	1 (14 yr-o)	1	Duration of support: 179 days Bridged to transplant: Yes	Hemorrhage Pump thrombus/fibrin Desensitization therapy	1	0
Chu et al. (49)	Berlin Heart VAD	1 (4 yr-o)	1	Duration of support: 13 days	Fibrin collection on valve Influenza Metabolic acidosis Peritonitis Diffuse bowel necrosis Multifocal thrombi in LCA	0	1
Jabbar et al. (54)	HeartMate II	1 (23 yr-o)	1	Duration of support: 3 yrs 1 mth Bridged to transplant: Yes	Stroke Neurological complications	1	0
Shah et al. (55)	HeartMate II	6 adults	2 (44, 23 yr-o)	Duration of support: 493 and 261 days	Respiratory failure Decreased O_2_ Acute kidney injury Ventricular tachycardia	1	1
Morales et al. (56)	HeartMate II	1 (15 yr-o)	1	Duration of support: 72 days Bridge to transplant: yes CVP: 19 mmHg	None reported	1	0
O'Connor et al. (57, 58)	HeartMate III	35 peds	5	Bridged to transplant: 20 Remain alive on device: 13 Ventricular recovery: 1	Right heart failure Bleeding Seizures Infection	34	1
Frazieret al. (59)	HeartMate LVAS	1 (14 yr-o)	1	Duration of support: 45 days Bridged to transplant: yes	None reported	1	0
Woods et al. (60)	HeartWare HVAD TAH	1 (25 yr-o)	1	Duration of support: 1 month, 10 days Duration of support: waiting for donor	Hepatic failure Renal failure Vasoplegic Cerebral edema Cerebral herniation	0	1
Imielski et al. (61)	HeartWare HVAD	3 peds	3	Duration of support: ≤ 272 days Bridge to transplant: 3 ΔCVP: pre 15–29 post 9–22 mmHg	Collateral vessel coiling Pancreatitis Worsening HF Ascites Slurred speech	3	0
Tanoue et al. (62)	Jarvik 2000	1 (13 yr-o)	1	Duration of support: 3 years ΔCVP: 6 mmHg	None reported	1	0
Rossano et al. (63)	TAH^*^	1 (13 yr-o)	1	Duration of support: 61 days Bridged to transplant: yes	Plastic bronchitis Renal failure Bleeding Stent placement	1	0
Newcomb et al. (64)	Thoratec extracorporeal VAD (also make HeartMate)	1 (25 yr-o)	1	Duration of support: 5 mths Bridged to transplant: yes ΔCVP: 7 mmHg	None reported	1	0

* *70-cc Syncardia total artificial heart (SynCardia Systems, Inc, Tucson, AZ). °9 stage I, 12 stage II, 5 stage III; 1 stage I, 7 stage II, 3 stage III*.

In addition to the BH, many mechanical assist devices approved by the FDA for use in adults with heart failure have been used off-label in the pediatric population. Data regarding the HeartMate (HM) II in a Fontan circulation is limited to case studies, lowering one patient's CVP levels by 19 mmHg (Table 1) (56, 59). The HM II has also supported young adults with a failed Fontan circulation for up 3 years before being bridged to transplantation (54, 55). A recently published report of 35 patients with pediatric heart failure, 5 with a Fontan circulation, who received a HM III since December 2017 shows encouraging results (Table 1) (57, 58). While detailed data relative to each patient's congenital defect are not available, of the 35 patients only 1 had died at the time of paper submission in September 2019 (57). Further, 20 patients were successfully bridged to transplantation, 13 remain alive on the device, and one patient experienced ventricular recovery and was weaned off the device (57). This study represents the highest survival rate for the longest period of time in a pediatric cohort receiving a long-term mechanical device. However, seven patients experienced elevated CVP levels >16 mmHg and exhibited clinical manifestations of high CVP while supported by the HM III, including edema and hepatic deterioration (57). Thus, this long-term mechanical assist device successfully bridges pediatric patients to transplant but does not ultimately address all cases of high CVP.

Off-label use of several other mechanical assist devices intended for adult heart failure (HF) are noted in Table 1, including the HeartWare, the Jarvik 2000, the SynCardia Total Artificial Heart, and the Thoratec VAD (predecessor of HeartMate) (52, 53, 60–64). These devices have seen scarce use in patients with a failed Fontan circulation and are limited to case studies, making it difficult to draw conclusions on their effectiveness within the Fontan population.

### Mechanical Support Devices Under Development for Patients With Fontan Failure

Several groups have identified the need for a mechanical assist device that does not assist the systemic ventricle in hopes of improving CVP, but rather is implanted in the central venous system. All groups (Table 2) tested these devices in an *in vitro* system, with tunable capacitance and resistance, representative of Fontan physiology (65, 67, 69, 70). The largest *in vitro* change in pressure (7.6 mmHg) was found by Rodefeld et al. using a derivative of the Von Karman viscous rotary pump (Table 2) (66, 67). However, the pump was designed for placement in a conduit parallel to the Fontan connection, introducing additional surgical considerations and challenges (67). An artificial right ventricle (ARV) developed by researchers at the University of Colorado found an estimated 5 mmHg pressure change in CVP (Table 2) (68). The drawback to their device design is the use of an external power source rather than remotely powering the ARV. This device also requires implantation of a bifurcated conduit, restricting its application to a specific Fontan geometry (68). On the contrary, it may be beneficial to have a venous assist device in a conduit separate from the Fontan connection to decrease the risk associated with pump failure and obstruction. It is important to note that the Von Karman derivative and the ARV were not tested under pulsatile conditions (65, 67, 68). One group did in fact test their aortic turbine venous assist pump (iATVA) in a pulsatile environment, which introduced a host of limitations related to pulse propagation throughout the system and pump instability (Table 2) (67). This study magnifies the necessity for pulsatile *in vitro* systems in order to properly test circulatory mechanical assist devices. Thus, more accurate testing and design modifications are required to determine the efficiency of the aforementioned devices within a Fontan circulation.

**Table 2:** Mechanical support devices under development for implantation within the total cavopulmonary connection in patients with Fontan circulation in an effort to assist pulmonary flow.

**References**	**Cardiac support device**	**Implant location**	**Testing method**	**Outcome measure**	**Limitations**
Rodefeld et al. (65, 66)	Von Karman viscous rotary pump with remote power	Intersection of PA, IVC, SVC	*In vitro* mock Fontan circulation with modular resistance and capacitance	Flow: 5 L/min ΔP: 7.6 mmHg CO increase: 12.8%	- Large power draw at high RPMs - No thrombus formation or hemolytic testing
Pekkan et al. (67)	Aortic turbine venous assist (iATVA) with remote power and rotating impeller	Parallel to IVC in bypass line	*In vitro* pulsatile single ventricle circulation; modular resistance and capacitance	Flow: 3.2 L/min ΔP: 2–3 mmHg Aortic flow steal: 1 L/min	- Patients must be 20 kg - Pulsatile conditions cause pump/IVC pulsatility - Turbine flow limited to 0.9 L/min - Optimization of friction and pump leakage
Lacour-Gayet et al. (68)	Artificial right ventricle (ARV) with rotating impeller and external power source	Pulmonary trunk (b/t LPA/RPA and IVC/SVC)	*In vitro* Fontan circulation modular resistance and capacitance	ΔCVP: 5 mmHg Added CO: 2 L/min ΔP across ARV: 2 mmHg at 4L/min	- External power source - No thrombus formation or hemolytic testing - No systemic circulation - Controlled pulmonary afterload
Granegger et al. (69)	Double suction pump with single impeller	Intersection of PA/IVC/SVC	*In vitro* junction of PA/IVC/SVC and virtual implantation	ΔP across pump: 2.5 mmHg Power loss: 3 W	- External power source - Fontan patients 10+ years - Computational assumptions (boundary conditions, simplified eqns for associations, no friction)
Cysyk et al. (70)	Three blade rotors with embedded motor controller	Between IVC/SVC to PA	*In vitro* mock Fontan circulation and *in vivo* sheep model (1 sheep, 30 days)	*In vivo* sheep model ΔP across pump: 3 mmHg Δthromboplastin: 29 s Sheep survived till sacrifice	- Thrombi in PA outlet rotor - Outlet cannula bent and thrombi present - Increased thromboplastin - PA catheter migration into RV - Edema of chest

A group out of Penn State College of Medicine performed both *in vitro* and *in situ* experimentation of a venous assist device placed at the four way junction of the PA, IVC, and SVC (70). The *in vitro* work delivered promising results with an inverse relationship between pressure head and flow (70). The authors then implanted their device in a single sheep model for 30 days; post-operative complications included pressure catheter migration from the PA to the RV, edema, and increased levels of thromboplastin, a protein responsible for initiating blood coagulation (70). The explant data revealed a severely bent outlet cannula with multiple thrombi present and thrombus formation on the outlet rotor (70). This study confirms the importance of device testing in a large animal model with Fontan circulation for a better understanding of design needs and *in situ* function.

### Clinical Relevance

The performance of mechanical assist devices in patients with SVD has improved markedly since the days of ECMO, but the outcomes of the aforementioned studies demonstrate that problems persist when using these devices long-term. In many cases, implantation of an MSD introduced a host of related complications (thrombi, bleeding, stroke, etc.) without lowering CVP or curbing symptoms of circulatory dysfunction, which begs the question of whether or not using MSDs indefinitely is a plausible solution for the SVD population. Further, venous assist devices under development have experienced similar issues of thrombosis and this complication must be addressed prior to human trials. Based on the results discussed in this section, we believe an MSD is not the ideal candidate for long-term support of patients with a Fontan circulation due to the characteristic nature of heavy alloys and the inevitable hematological complications.

## Tissue Engineered and Stem-Cell Based Therapies for Preventing Fontan Failure

Tissue engineering was coined by Langer and Vacanti nearly 30 years ago as an interdisciplinary approach that utilizes cells, scaffolds, and bioactive molecules to develop biological substitutes for damaged tissues (71). Since its inception, the field has hosted a wide variety of applications. In particular, regeneration of damaged heart tissue has been of interest, most likely due to the high mortality rate associated with ischemic and congenital heart diseases. Although the cause of SVD is not entirely understood, studies indicate abnormalities in cardiomyocyte signaling, replication, and proliferation during fetal heart development contribute to defect formation (72–74). Additionally, there has been evidence of human heart regeneration (75, 76), and the discovery of these mechanisms has encouraged the use of stem-cells in congenital heart defect therapies. Herein, we discuss tissue engineering and its subcomponents as it applies to prevention of RV failure and inadequate Fontan circulation.

### Stem Cell Therapies for Preventing Fontan Failure

Many cell types have been used for tissue engineering purposes, but a subset of stem cell lineages have proven most effective at improving ventricular function in patients with SVD (Table 3). Cardiac progenitor cells, or stem cells that have committed to a cardiac fate, were first delivered to patients with HLHS in the Transcoronary Infusion of Cardiac Progenitor Cells in Patients With Single Ventricle Physiology (TICAP) clinical trial (77). Fourteen patients received autologous cardiosphere-derived cells (CDCs) delivered via catheterization 1 month after stage I or stage II palliation. Compared to the control group, patients that received CDCs displayed improved RV ejection fraction (mean 31.5 vs. 40.4%) 18 months after delivery and no adverse events were reported, thus demonstrating safety and efficacy (77). After 36 months, the CDC group had a significantly higher cardiac index compared to the control group (mean 4.9 vs. 3.6 L/min/m^2^), maintained superior RV ejection fraction (mean 41.8 vs. 32.3%), and exhibited a larger absolute change in RV ejection fraction (mean +5.7% and −2.2%) (78). Following these promising results, researchers moved to a randomized phase II clinical trial called Cardiac Progenitor Cell Infusion to Treat Univentricular Heart Disease, or PERSEUS. This clinical trial enrolled 41 eligible patients, 17 of which were randomly allocated to the control group and later had the option of receiving CDC infusion (79). The 34 patients that initially received CDC infusion demonstrated significant improvements in ejection fraction after 3 months compared to baseline values (mean 41.7 vs. 35.3%) (79). A year after treatment, patients maintained improved cardiac function, with an absolute change in ejection fraction from baseline to +7.4% (79). Researchers then began multicenter, randomized phase III clinical trial in 2016 coined Cardiac Stem/Progenitor Cell Infusion in Univentricular Physiology (APOLLON Trial: NCT02781922). No results have been published yet for this trial.

**Table 3:** Clinical trials that have/are using stem cell therapies for prevention and recovery of the failed Fontan circulation.

**NCT identification number**	**Name**	**Abbreviation**	**Cell Type**	**Target therapy**	**Dates**
01273857	Transcoronary infusion of cardiac progenitor cells in patients with single ventricle physiology	TICAP	CDCs	Prevention	January 11, 2011 to June 15, 2015
01829750	Cardiac progenitor cell infusion to treat univentricular heart disease	PERSEUS	CDCs	Prevention	April 11, 2013 to January 5, 2017
02781922	Cardiac stem/Progenitor cell infusion in univentricular physiology	APOLLON	CDCs	Prevention	June 2016 to May 2023
01883076	Safety study of autologous umbilical cord blood cells for treatment of hypoplastic left heart syndrome	N/A	UCB MSCs	Prevention	May 15 2013 to November 2020 (extended May 2021)
03525418 (previously 02398604)	Allogenic hMSC injection in patients with HLHS	ELPIS	hMSCs	Prevention	March 25, 2015 to June 25, 2019
02549625	Safety and feasibility study of autologous bone marrow derived mononuclear cells	N/A	BM -MNCs	Recovery	August 2015 to April 2021

In addition to cardiac progenitor cells, several groups have investigated the benefit of mesenchymal stem cells (MSCs) on systemic ventricular function in patients with SVD (Table 3). MSCs are multipotent and can be found in several tissues throughout the body, including bone marrow, adipose tissue, umbilical cord blood (UCB), and the placenta (80). The Mayo Clinic reported the first case study of a 4 months old infant with HLHS to receive UCB derived stem cells via a manufactured cell product (81). This product contains a high concentration of autologous mononuclear cells that cannot be obtained using traditional UCB cell isolation methods, and is delivered directly to the operating room where it is thawed and administered (82). Cells were delivered intraoperatively during stage II palliation near the apex of the right ventricle with no adverse events. At 3 months, the patient's RV ejection fraction improved by roughly 15% and a 29% fractional area change was observed (all compared to baseline) (81). Researchers followed with a phase I, non-randomized clinical trial to determine safety and feasibility of autologous UCB stem cell injection in the RV of patients with HLHS. Ten patients received treatment during the stage II palliation and no adverse events were reported until 3 months after treatment; one patient died due to multiorgan failure after elective colostomy (82). Although this late mortality was not associated with UCB injection, the study was placed on hold for further review by the Data and Safety Monitoring Board (82). No additional results have been published from this clinical trial, though it remains active and is expected to complete in November 2020 (NCT01883076).

Lastly, a phase I/IIb clinical trial named Allogenic hMSC Injection in Patients With HLHS (ELPIS) began in March 2015 at the University of Maryland. The study was designed to enroll 30 patients with HLHS, beginning with 10 patient injections during stage II palliation to ensure safety, followed by 20 patient injections to determine efficacy of improved RV function (83). However, only five patients were enrolled, and the trial was prematurely terminated due to the sponsor's move to the private sector (NCT03525418).

### Tissue Engineered Therapies for Preventing Fontan Failure

Tissue engineered conduits for use in the Fontan-Kreutzer procedure are being studied to replace commercial, non-living grafts. A pediatric surgeon, Toshiharu Shin'oka, implanted the first autologous engineered grafts in SVD patients during a 2001 Japanese clinical trial (84). Midterm results showed no adverse events for the 23 patients, but late term results indicated stenosis within the graft in several children (84, 85). Similar findings were discovered in the first United States clinical trial at Nationwide Children's Hospital, where 25% of patients experienced stenosis (85, 86). However, both studies demonstrated exceptional graft growth and they've found that graft patency can be maintained by delivering cells to the scaffold in a dose-dependent manner (87, 88). These studies demonstrate an autologous tissue engineered graft with the ability to remodel and grow with the host environment, a revolutionary finding for patients undergoing the Fontan-Kreutzer procedure. However, this solution does not address the long-term complications associated with passive flow and congestion within the Fontan circuit.

A group out of Tokyo Women's Medical University utilized cell sheet technology, a method in which cells are grown in a temperature sensitive dish and are released as a sheet when exposed to low temperatures, for application in the Fontan circulation (89, 90). This technology has also been harnessed to regenerate adult tissue following ischemic myocardial events and the results indicate cell sheet technology is effective in improving ventricular ejection fraction, end diastolic dimensions, pulmonary pressures, and exercise tolerance (91). Shimizu et al. cultured cardiomyocytes derived from human induced pluripotent stem cells (hiPSCs) and wrapped two triple-layered cell sheets around the IVC of 13 nude rats (90). Spontaneous beating (independent of host cardiac pulsation) was observed 3 weeks after implantation, but inner pressure changes were minimal and not significantly different between 4 and 8 weeks timepoints (median 0.05 mmHg vs. 0.27 mmHg) (90). Pacing and engraftment failure were also apparent in a small portion of rats at each time point (90). This group is now developing a new method to combine hiPSC derived cardiomyocytes with human dermal fibroblasts to increase maturation of cardiomyocytes, and thus better recapitulate native myocardium function, *in vitro* (92).

Similar to the previous study mentioned, a group at the University Heart Center Hamburg in Germany developed engineered heart tissues (EHTs) using neonatal rat cardiomyocytes, in combination with biomaterials, and tested them in the vena cava of male rats (93). Two EHTs, each 6–8 mm in length and 1 mm in diameter, were wrapped around the dissected SVC of 12 rats and sacrificed at 7, 14, 28, and 56 days for analyses (93). Upon implantation, EHTs were contractile and after explantation, the structures maintained microscopic contractility. Histology revealed dense vascularization, including evidence of capillaries and differentiated cardiomyocytes with advanced organization (93). The authors note that this is a proof-of-concept study; however, no follow-up studies have been published since this 2016 paper. In addition, the method for evaluating conduit function had many drawbacks; there was no demonstration of flow through the construct and the hemodynamic evaluation relied on cross-clamping for significant changes in pressure.

More recently, a report out of Yale University details the design and *in vitro* performance of a modular tissue engineered pulsatile conduit (TEPC) (94). Briefly, the conduit base is a decellularized human umbilical artery (HUA) seeded with human cardiac fibroblasts. The base is wrapped in an engineered heart tissue composed of decellularized porcine heart matrix, human stem cell derived cardiomyocytes, and adult human cardiac fibroblasts (94). This design proved superior in compliance and cardiomyocyte contractility compared to other designs tested by the authors, including strategies with a polyglycolic acid mesh and a collagen-based scaffold in place of decellularized porcine heart matrix (94). The TEPC was tested using a mechanical perfusion bath equipped with field stimulating electrodes. The perfusion chamber and inner lumen were filled with Tyrode solution, and the inner lumen of the TEPC was held constant at 10 mmHg. Without electrical stimulation, spontaneous beating was apparent and resulted in an average 0.68 mmHg change in pressure. When electrically stimulated at 1 and 2 Hz, the recorded internal pressure changes were, on average, 0.75 and 0.83 mmHg (94). This study represents the most advanced tissue engineered structure, in both testing and design, for prevention of a failed Fontan circuit. While this study was published to support the authors' TEPC design and conceptualization, further studies are required to better understand its clinical application to the Fontan circulation. Future TEPC design considerations should include switching to an autologous cell source, testing alternatives to HUA, and robust mechanical and electrical stimulation for cardiomyocyte maturation and thus increased pulse pressures.

### Clinical Relevance

The caveat in determining the clinical relevance of the discussed tissue engineered solutions in *preventing* Fontan circulation failure is discerning the impact of early intervention on late outcomes. While it is evident that both MSCs and CDCs improve single ventricle function short-term, it would be extremely difficult to ascertain the effect that each cell type has on late Fontan failure years after intervention, especially in patients where HF symptoms stray from the classic phenotype. A main takeaway from the MSC and CDC based clinical trials, though, is the safety and efficacy of autologous stem cell use within the SVD population, and the ability to modulate tissue engineered structures by delivering cells to a scaffold in doses. This information has undoubtedly informed the tissue engineering community working toward Fontan failure therapies, which is evident in the design of each solution discussed here. We strongly support a push toward human induced pluripotent stem cells as the most scalable and efficient path forward for such therapies, and we recommend the use of scaffolds for cell retention purposes. In order to properly evaluate and compare said therapies, a standardized *in vitro* protocol would be invaluable and accelerate the development of a tissue engineered solution for preventing Fontan failure.

## Tissue Engineered and Stem Cell-Based Therapies for Repairing Fontan Failure

Very few groups have utilized tissue engineering for reversal of Fontan failure, as the risk of imminent death is high once symptoms present, and alternative rescue methods are well-understood and fairly reliable (i.e., mechanical assist). The following section discusses the few case studies and research groups that have used tissue engineering strategies for repairing circulatory failure in children with Fontan physiology.

### Stem Cell Therapies for Repairing Fontan Failure

One clinical trial to date has examined the benefit of MSCs on declining ventricle function after Fontan palliation (NCT02549625). The cells utilized in this trial were bone marrow derived mononuclear cells (BM-MNCs), which contain a heterogenous population of cells, including MSCs, and regenerative paracrine factors, delivered via an intracoronary catheter (95). This phase I trial enrolled 10 patients and is expected to complete in April 2021. The primary outcome measure of the study was the number of related serious adverse events after cell delivery while the secondary outcome included monitoring changes related to cardiac structure and function; both were monitored for 2 years.

The aforementioned clinical trial has published a report detailing infusion of BM-MNCs in the right and left coronary arteries of a 25 years old man with Fontan circulation, an ejection fraction of 34%, severe ascites, aortic valve regurgitation, and a dilated RV (95). The patient was a poor candidate for cardiac transplantation due to desensitization to human leukocyte antigen and had received consistent outpatient therapy with little sustained resolution, making him an obvious candidate for this study (95). He experienced no adverse events during surgery or at the 1, 3, and 6 months follow-up visits (although he had one episode of palpitations) (95). The patient experienced the maximal therapeutic effect at 3 months, exhibiting an estimated RV EF of 45%, although it declined to 38% at 6 months (95). This case demonstrates the first intracoronary infusion of stem cells in a patient with Fontan circulation and HF symptoms. While this case study provides promising anecdotal evidence that BM-MNCs have a benefit on the failing systemic ventricle, additional cases must be presented to ensure efficacy and safety of this therapeutic, which are expected upon completion of the clinical trial (NCT02549625).

An early case report from Giessen, Germany details a patient with declining ventricle function after stage II palliation for correction of HLHS (96). Chronic failure of the right ventricle after repeated resolution attempts led the family and clinicians to consider heart transplantation; however, doctors initiated a rescue stem cell therapy on a compassionate basis (96). This patient also received autologous BM-MNC treatment after isolation from an iliac spine aspirate, albeit within a Norwood circulation. The patient experienced no adverse events during or after intracoronary infusion of BM-MNCs, and displayed oxygen saturations up to 88% and an RV EF of 44% 3 months after infusion (96). These results encourage the use of BM-MNCs in the SVD population, although this is a single report. The phase I clinical trial to be completed in April 2021 will hopefully provide a better understanding of BM-MNCs as an avenue for reversal of a failed Fontan circulation.

### Tissue Engineered Therapies for Repairing Fontan Failure

Few research groups have targeted the SVD population as a recipient of a tissue engineered cardiac patch for improvement of the systemic ventricle. This may be due to the fragile nature and rapid decline of these patients, or the barriers presented when seeking approval from regulatory bodies. However, many groups have investigated the effect of tissue engineered structures for improvement of ventricle function in adults with heart failure (97–107). A collaboration between researchers at Georgia Institute of Technology (GIT) and the University of California San Diego (UCSD) launched the development of a cardiac patch geared toward pediatric patients experiencing heart failure (108). The three-dimensionally printed patch consists of autologous human cardiac progenitor cells, porcine cardiac extracellular matrix, and a synthetic biomaterial (gelatin methacrylate). It aims to release paracrine factors to the native myocardium that signal the host to regenerate cardiac tissue (108). Their most recently published work details the feasibility of patch implantation in a rat model. Vascularization was observed 14 days after implantation of the patch on the epicardium, indicating adequate nutrient delivery and integration with the host environment (108). Future studies will focus on efficacy of the patch in improving myocardial function. Although they have not tested this patch in patients with SVD, they have observed effectiveness in late ventricular failure in adults (107). These studies combined provide excellent understanding of patch composition relative to paracrine factor modulation and encourages the use of tissue engineering strategies for reversal of Fontan failure.

### Clinical Relevance

The studies detailed in this section highlight the usefulness of small molecules in signaling the damaged myocardium to regenerate. As was previously stated, BM-MNCs contain an array of paracrine molecules and progenitor cells, both of which have the ability to cause regeneration provided the appropriate scaffold. However, the topology, rigidity, and chemical makeup of the scaffold that progenitor cells are seeded onto or in can alter cell function and phenotype (109), indicating that for tissue engineered structures, the “perfect storm” of scaffold(s), cells, and signaling molecules must be used in order to obtain the desired therapeutic effect. A reasonable approach to expedite the clinical application of a tissue engineered structure for use in patients with SVD is exemplified by collaborators at GIT and UCSD. These researchers used an autologous stem cell source, which minimizes host rejection due to antibody mismatch, and a scaffold (porcine cardiac ECM) that would likely promote a cardiac cell fate. Finally, the cascade of signaling molecules triggered by factors released via this tissue engineered structure evidently encourage the desired therapeutic response, as shown by the results from the pediatric progenitor patch animal study and the phase I clinical trials in adults with late HF (107, 108).

## Conclusions

Families and patients face an arduous journey when diagnosed with SVD, involving years of palliative surgeries and impending patient decline. This population experiences Fontan failure in a variety of presentations, ranging from lymphatic to ventricular dysfunction, and are nearly always accompanied by high CVP. In addition, Fontan failure is multifactorial and arrythmia, cardiac failure, valvular incompetence, systemic outflow obstruction, desaturation due to arteriovenous malformations, and lymphatic congestion can all lead to raised CVP. These elevated pressures are likely responsible for multi-organ decline, but the pathogenesis is not entirely understood, making it difficult to develop targeted therapies.

Upon developing circulatory dysfunction, patients with SVD are destined for a heart transplant or death. In the event that neither is an option, MSDs are used until a suitable donor heart is available. While it seems that MSDs are able to support patients until transplantation, many still experience Fontan failure symptoms or problems associated with the MSD implantation. In the long-term, complications arise with MSDs and are most often related to blood irregularities, eliminating this as a viable solution for indefinite use.

Development of a tissue engineered structure for Fontan failure, both for the single ventricle and venous return, have developed significantly in the past several decades. However, researchers have taken various approaches, and this has slowed progress. Stem cells delivered to the ventricle show promising results but likely don't have a lasting effect years after they are administered, leading us to recommend the use of a scaffold for cell and structural retention. It is also unclear whether or not a tissue engineered patch on the single ventricle has the ability to lower CVP and the associated complications, as the absence of a pulmonary ventricle is likely responsible. A uniform animal model and *in vitro* testing method should include pulsatile flow and alike Fontan parameters in order to compare tissue engineered therapies. Ultimately, cell-based therapies have displayed improvements in cardiovascular function in patients with SVD and represents a promising area for further investigation.

## Author Contributions

JJ and MF: manuscript conception and design. MF: interpretation of relevant literature and drafting of manuscript. MD: medical expertise in single ventricle physiology. JJ and MD: critical manuscript revisions. MF and MD figure preparation. All authors contributed to the article and approved the submitted version.

## Conflict of Interest

The authors declare that the research was conducted in the absence of any commercial or financial relationships that could be construed as a potential conflict of interest.

## Abbreviations

RV: right ventricle
CVP: central venous pressure
SVD: single ventricle disease
CHD: congenital heart defect
HLHS: Hypoplastic Left Heart Syndrome
BT: Blalock-Taussig
PA: pulmonary artery
SVC: superior vena cava
IVC: inferior vena cava
PLE: protein losing enteropathy
PB: plastic bronchitis
FALD: Fontan-associated liver disease
MSD: mechanical support device
ECMO: extracorporeal membrane oxygenation
BH: Berlin Heart
VAD: ventricular assist device
HM: HeartMate
FDA: Food and Drug Administration
HDE: humanitarian device exemption
HF: heart failure
ARV: artificial right ventricle
iATVA: aortic turbine venous assist pump
CDC: cardiosphere-derived cell
MSC: mesenchymal stem cell
UCB: umbilical cord blood
hiPSC: human induced pluripotent stem cell
EHT: engineered heart tissue
TEPC: tissue engineered pulsatile conduit
HUA: human umbilical artery
BM-MNC: bone marrow derived mononuclear cell
GIT: Georgia Institute of Technology
UCSD: University of California San Diego
ECM: extracellular matrix
TAH: total artificial heart
HT: heart transplant
LCA: left coronary artery.

## References

[1] Kritzmire SM Cossu AE. Hypoplastic Left Heart Syndrome. Treasure Island (FL)2020. 32119463

[2] Rychik J. Forty years of the Fontan operation: a failed strategy. Semin Thorac Cardiovasc Surg Pediatr Card Surg Annu. (2010) 13:96–100. 10.1053/j.pcsu.2010.02.006 20307870

[3] Norwood WI Lang P Hansen DD. Physiologic repair of aortic atresia-hypoplastic left heart syndrome. N Engl J Med. (1983) 308:23–6. 10.1056/NEJM198301063080106 6847920

[4] Sano S Ishino K Kawada M Arai S Kasahara S Asai T. Right ventricle-pulmonary artery shunt in first-stage palliation of hypoplastic left heart syndrome. J Thorac Cardiovasc Surg. (2003) 126:504–9; discussion 9–10. 10.1016/S0022-5223(02)73575-7 12928651

[5] Glenn WW Ordway NK Talner NS Call EP Jr. Circulatory bypass of the right side of the heart. vi. shunt between superior vena cava and distal right pulmonary artery; report of clinical application in thirty-eight cases. Circulation. (1965). 31:172–89. 10.1161/01.CIR.31.2.172 14261735

[6] Gewillig M Brown SC Heying R Eyskens B Ganame J Boshoff DE. Volume load paradox while preparing for the Fontan: not too much for the ventricle, not too little for the lungs. Interact Cardiovasc Thorac Surg. (2010) 10:262–5. 10.1510/icvts.2009.218586 19945986

[7] McElhinney DB Petrossian E Reddy VM Hanley FL. Extracardiac conduit Fontan procedure without cardiopulmonary bypass. Ann Thorac Surg. (1998) 66:1826–8. 10.1016/S0003-4975(98)00928-X 9875809

[8] Gewillig M. The Fontan circulation. Heart. (2005) 91:839–46. 10.1136/hrt.2004.051789 15894794 PMC1768934

[9] Hebson CL McCabe NM Elder RW Mahle WT McConnell M Kogon BE. Hemodynamic phenotype of the failing Fontan in an adult population. Am J Cardiol. (2013) 112:1943–7. 10.1016/j.amjcard.2013.08.023 24075283 PMC4505550

[10] Feldt RH Driscoll DJ Offord KP Cha RH Perrault J Schaff HV. Protein-losing enteropathy after the Fontan operation. J Thorac Cardiovasc Surg. (1996) 112:672–80. 10.1016/S0022-5223(96)70051-X 8800155

[11] Caruthers RL Kempa M Loo A Gulbransen E Kelly E Erickson SR. Demographic characteristics and estimated prevalence of Fontan-associated plastic bronchitis. Pediatr Cardiol. (2013) 34:256–61. 10.1007/s00246-012-0430-5 22797520 PMC3586576

[12] Wu FM Ukomadu C Odze RD Valente AM Mayer JE Jr Earing MG. Liver disease in the patient with Fontan circulation. Congenit Heart Dis. (2011) 6:190–201. 10.1111/j.1747-0803.2011.00504.x 21443554

[13] Levitt DG Levitt MD. Protein losing enteropathy: comprehensive review of the mechanistic association with clinical and subclinical disease states. Clin Exp Gastroenterol. (2017) 10:147–68. 10.2147/CEG.S136803 28761367 PMC5522668

[14] Rychik J Atz AM Celermajer DS Deal BJ Gatzoulis MA Gewillig MH. Evaluation and management of the child and adult with fontan circulation: a scientific statement from the american heart association. Circulation. (2019) 2019:CIR0000000000000696. 10.1161/CIR.0000000000000696 31256636

[15] Allen KY Downing TE Glatz AC Rogers LS Ravishankar C Rychik J. Effect of Fontan-associated morbidities on survival with intact Fontan circulation. Am J Cardiol. (2017) 119:1866–71. 10.1016/j.amjcard.2017.03.004 28385177

[16] Arcidi JM Jr Moore GW Hutchins GM. Hepatic morphology in cardiac dysfunction: a clinicopathologic study of 1000 subjects at autopsy. Am J Pathol. (1981) 104:159–66. 6455066 PMC1903755

[17] Asrani SK Warnes CA Kamath PS. Hepatocellular carcinoma after the Fontan procedure. N Engl J Med. (2013) 368:1756–7. 10.1056/NEJMc1214222 23635071

[18] Greenway SC Crossland DS Hudson M Martin SR Myers RP Prieur T. Fontan-associated liver disease: Implications for heart transplantation. J Heart Lung Transplant. (2016) 35:26–33. 10.1016/j.healun.2015.10.015 26586487

[19] Collins RT 2nd Doshi P Onukwube J Fram RY Robbins JM. Risk factors for increased hospital resource utilization and in-hospital mortality in adults with single ventricle congenital heart disease. Am J Cardiol. (2016) 118:453–62. 10.1016/j.amjcard.2016.05.020 27291967

[20] Pundi KN Johnson JN Dearani JA Pundi KN Li Z Hinck CA. 40-year follow-up after the fontan operation: long-term outcomes of 1,052 patients. J Am Coll Cardiol. (2015). 66:1700–10. 10.1016/j.jacc.2015.07.065 26449141

[21] Gamba A Merlo M Fiocchi R Terzi A Mammana C Sebastiani R. Heart transplantation in patients with previous Fontan operations. J Thorac Cardiovasc Surg. (2004) 127:555–62. 10.1016/j.jtcvs.2003.08.016 14762368

[22] Michielon G Parisi F Squitieri C Carotti A Gagliardi G Pasquini L. Orthotopic heart transplantation for congenital heart disease: an alternative for high-risk fontan candidates? Circulation. (2003) 108 (Suppl. 1):II140–9. 10.1161/01.cir.0000087442.82569.51 12970223

[23] Kulkarni A Patel N Singh TP Mossialos E Mehra MR. Risk factors for death or heart transplantation in single-ventricle physiology (tricuspid atresia, pulmonary atresia, and heterotaxy): A systematic review and meta-analysis. J Heart Lung Transplant. (2019) 38:739–47. 10.1016/j.healun.2019.04.001 31006521

[24] Bernstein D Naftel D Chin C Addonizio LJ Gamberg P Blume ED. Outcome of listing for cardiac transplantation for failed Fontan: a multi-institutional study. Circulation. (2006) 114:273–80. 10.1161/CIRCULATIONAHA.105.548016 16847155

[25] Lamour JM Kanter KR Naftel DC Chrisant MR Morrow WR Clemson BS. The effect of age, diagnosis, and previous surgery in children and adults undergoing heart transplantation for congenital heart disease. J Am Coll Cardiol. (2009) 54:160–5. 10.1016/j.jacc.2009.04.020 19573734

[26] Almond CSD Thiagarajan RR Piercey GE Gauvreau K Blume ED Bastardi HJ. Waiting list mortality among children listed for heart transplantation in the United States. Circulation. (2009) 119:717–27. 10.1161/CIRCULATIONAHA.108.815712 19171850 PMC4278666

[27] Hoganson DM Boston US Gazit AZ Canter CE Eghtesady P. Successful bridge through transplantation with berlin heart ventricular assist device in a child with failing fontan. Ann Thorac Surg. (2015) 99:707–9. 10.1016/j.athoracsur.2014.04.064 25639417

[28] Etz C Welp H Tjan TD Krasemann T Schmidt C Scheld HH. Successful long-term bridge to transplant in a 5-year-old boy with the EXCOR left ventricular assist device. Thorac Cardiovasc Surg. (2004) 52:232–4. 10.1055/s-2004-821017 15293161

[29] Calvaruso DF Ocello S Salviato N Guardi D Petruccelli DF Rubino A. Implantation of a Berlin Heart as single ventricle by-pass on Fontan circulation in univentricular heart failure. ASAIO J. (2007) 53:e1–2. 10.1097/MAT.0b013e31815a2500 18043136

[30] Mackling T Shah T Dimas V Guleserian K Sharma M Forbess J. Management of single-ventricle patients with Berlin Heart EXCOR Ventricular Assist Device: single-center experience. Artif Organs. (2012) 36:555–9. 10.1111/j.1525-1594.2011.01403.x 22236151

[31] Pretre R Haussler A Bettex D Genoni M. Right-sided univentricular cardiac assistance in a failing Fontan circulation. Ann Thorac Surg. (2008) 86:1018–20. 10.1016/j.athoracsur.2008.03.003 18721610

[32] Almond CS Singh TP Gauvreau K Piercey GE Fynn-Thompson F Rycus PT. Extracorporeal membrane oxygenation for bridge to heart transplantation among children in the United States: analysis of data from the organ procurement and transplant network and extracorporeal life support organization registry. Circulation. (2011) 123:2975–84. 10.1161/CIRCULATIONAHA.110.991505 21670232

[33] Ford MA Gauvreau K McMullan DM Almodovar MC Cooper DS Rycus PT. Factors associated with mortality in neonates requiring extracorporeal membrane oxygenation for cardiac indications: analysis of the extracorporeal life support organization registry data. Pediatr Crit Care Med. (2016) 17:860–70. 10.1097/PCC.0000000000000842 27355824

[34] Kumar TK Zurakowski D Dalton H Talwar S Allard-Picou A Duebener LF. Extracorporeal membrane oxygenation in postcardiotomy patients: factors influencing outcome. J Thorac Cardiovasc Surg. (2010) 140:330–6 e2. 10.1016/j.jtcvs.2010.02.034 20637917

[35] Allan CK Thiagarajan RR del Nido PJ Roth SJ Almodovar MC Laussen PC. Indication for initiation of mechanical circulatory support impacts survival of infants with shunted single-ventricle circulation supported with extracorporeal membrane oxygenation. J Thorac Cardiovasc Surg. (2007) 133:660–7. 10.1016/j.jtcvs.2006.11.013 17320562

[36] Griselli M Sinha R Jang S Perri G Adachi I. Mechanical Circulatory Support for Single Ventricle Failure. Front Cardiovasc Med. (2018) 5:115. 10.3389/fcvm.2018.00115 30211172 PMC6122112

[37] Miller JR Lancaster TS Callahan C Abarbanell AM Eghtesady P. An overview of mechanical circulatory support in single-ventricle patients. Transl Pediatr. (2018) 7:151–61. 10.21037/tp.2018.03.03 29770296 PMC5938256

[38] Woods RK Ghanayem NS Mitchell ME Kindel S Niebler RA. Mechanical circulatory support of the fontan patient. Semin Thorac Cardiovasc Surg Pediatr Card Surg Annu. (2017) 20:20–7. 10.1053/j.pcsu.2016.09.009 28007060

[39] Horne D Conway J Rebeyka IM Buchholz H. Mechanical circulatory support in univentricular hearts: current management. Semin Thorac Cardiovasc Surg Pediatr Card Surg Annu. (2015) 18:17–24. 10.1053/j.pcsu.2015.02.002 25939838

[40] Stiller B Weng Y Hubler M Lemmer J Nagdyman N Redlin M. Pneumatic pulsatile ventricular assist devices in children under 1 year of age. Eur J Cardiothorac Surg. (2005) 28:234–9. 10.1016/j.ejcts.2005.04.023 15949952

[41] Almond CS Buchholz H Massicotte P Ichord R Rosenthal DN Uzark K. Berlin Heart EXCOR Pediatric ventricular assist device Investigational Device Exemption study: study design and rationale. Am Heart J. (2011) 162:425–35 e6. 10.1016/j.ahj.2011.05.026 21884857

[42] Pace Napoleone C Cascarano MT Deorsola L Valori A. Ventricular assist device in a failing total cavopulmonary connection: a new step-by-step approach. Interact Cardiovasc Thorac Surg. (2018) 26:341–2. 10.1093/icvts/ivx288 29049683

[43] VanderPluym CJ Rebeyka IM Ross DB Buchholz H. The use of ventricular assist devices in pediatric patients with univentricular hearts. J Thorac Cardiovasc Surg. (2011) 141:588–90. 10.1016/j.jtcvs.2010.06.038 20692001

[44] VanderPluym CJ Khoo NS Rebeyka IM Buchholz H. Unique case of total artificial cardiac support in failed Fontan circulation after cardiectomy: is continuous flow better than pulsatile flow? J Thorac Cardiovasc Surg. (2013) 145:e62–3. 10.1016/j.jtcvs.2013.02.031 23518417

[45] Seitz S Buchholz H Rebeyka I Ross D West L Urschel S. Mechanical ventricular assist device as a bridge to recovery post-ABO-incompatible heart transplantation for failed Fontan circulation. Transpl Int. (2014) 27:e54–7. 10.1111/tri.12294 24628869

[46] Nathan M Baird C Fynn-Thompson F Almond C Thiagarajan R Laussen P. Successful implantation of a Berlin heart biventricular assist device in a failing single ventricle. J Thorac Cardiovasc Surg. (2006) 131:1407–8. 10.1016/j.jtcvs.2006.02.015 16733184

[47] Halaweish I Ohye RG Si MS. Berlin heart ventricular assist device as a long-term bridge to transplantation in a Fontan patient with failing single ventricle. Pediatr Transplant. (2015) 19:E193–5. 10.1111/petr.12607 26408232

[48] Cardarelli MG Salim M Love J Simone S Tumulty J Conway D. Berlin heart as a bridge to recovery for a failing Fontan. Ann Thorac Surg. (2009) 87:943–6. 10.1016/j.athoracsur.2008.07.086 19231431

[49] Chu MW Sharma K Tchervenkov CI Jutras LF Lavoie J Shemie SD Berlin Heart ventricular assist device in a child with hypoplastic left heart syndrome. Ann Thorac Surg. (2007) 83:1179–81. 10.1016/j.athoracsur.2006.08.020 17307489

[50] Pearce FB Kirklin JK Holman WL Barrett CS Romp RL Lau YR Successful cardiac transplant after Berlin Heart bridge in a single ventricle heart: use of aortopulmonary shunt as a supplementary source of pulmonary blood flow. J Thorac Cardiovasc Surg. (2009) 137:e40–2. 10.1016/j.jtcvs.2008.02.044 19154881

[51] Weinstein S Bello R Pizarro C Fynn-Thompson F Kirklin J Guleserian K The use of the Berlin Heart EXCOR in patients with functional single ventricle. J Thorac Cardiovasc Surg. (2014) 147:697–704; discussion −5. 10.1016/j.jtcvs.2013.10.030 24290716

[52] Niebler RA Ghanayem NS Shah TK De La Rosa Bobke A Zangwill S Brosig C. Use of a HeartWare ventricular assist device in a patient with failed Fontan circulation. Ann Thorac Surg. (2014) 97:e115–6. 10.1016/j.athoracsur.2013.11.075 24694452

[53] Valeske K Yerebakan C Mueller M Akintuerk H. Urgent implantation of the Berlin Heart Excor biventricular assist device as a total artificial heart in a patient with single ventricle circulation. J Thorac Cardiovasc Surg. (2014) 147:1712–4. 10.1016/j.jtcvs.2014.01.012 24521952

[54] Jabbar AA Franklin WJ Simpson L Civitello AB Delgado RM 3rd Frazier OH. Improved systemic saturation after ventricular assist device implantation in a patient with decompensated dextro-transposition of the great arteries after the Fontan procedure. Tex Heart Inst J. (2015) 42:40–3. 10.14503/THIJ-13-3374 25873797 PMC4378042

[55] Shah NR Lam WW Rodriguez FH 3rd Ermis PR Simpson L Frazier OH. Clinical outcomes after ventricular assist device implantation in adults with complex congenital heart disease. J Heart Lung Transplant. (2013) 32:615–20. 10.1016/j.healun.2013.03.003 23540399

[56] Morales DL Adachi I Heinle JS Fraser CD Jr. A new era: use of an intracorporeal systemic ventricular assist device to support a patient with a failing Fontan circulation. J Thorac Cardiovasc Surg. (2011). 142:e138–40. 10.1016/j.jtcvs.2011.05.018 21762934

[57] O'Connor MJ Lorts A Davies RR Fynn-Thompson F Joong A Maeda K. Early experience with the HeartMate 3 continuous-flow ventricular assist device in pediatric patients and patients with congenital heart disease: a multicenter registry analysis. J Heart Lung Transplant. (2020). 39:573–9. 10.1016/j.healun.2020.02.007 32111350

[58] Lorts A Villa C Riggs KW Broderick J Morales DLS. First use of heartmate 3 in a failing fontan circulation. Ann Thorac Surg. (2018). 106:e233–e4. 10.1016/j.athoracsur.2018.04.021 29752919

[59] Frazier OH Gregoric ID Messner GN. Total circulatory support with an LVAD in an adolescent with a previous Fontan procedure. Tex Heart Inst J. (2005) 32:402–4. 16392230 PMC1336720

[60] Woods RK Niebler R Kindel S Troshynski T Joyce LD Hraska V. A new method for implanting a total artifical heart in the patient with a Fontan circulation. J Thorac Cardiovasc Surg. (2019) 157:353–5. 10.1016/j.jtcvs.2018.08.082 30360935

[61] Imielski BR Niebler RA Kindel SJ Woods RK. Heartware ventricular assist device implantation in patients with fontan physiology. Artif Organs. (2017) 41:40–6. 10.1111/aor.12852 28093805

[62] Tanoue Y Fujino T Tatewaki H Shiose A. Jarvik 2000 axial flow ventricular assist device in right single ventricle after Fontan operation. J Artif Organs. (2019) 22:338–40. 10.1007/s10047-019-01124-4 31392523

[63] Rossano JW Goldberg DJ Fuller S Ravishankar C Montenegro LM Gaynor JW. Successful use of the total artificial heart in the failing Fontan circulation. Ann Thorac Surg. (2014) 97:1438–40. 10.1016/j.athoracsur.2013.06.120 24694426

[64] Newcomb AE Negri JC Brizard CP d'Udekem Y. Successful left ventricular assist device bridge to transplantation after failure of a fontan revision. J Heart Lung Transplant. (2006) 25:365–7. 10.1016/j.healun.2005.05.022 16507435

[65] Rodefeld MD Marsden A Figliola R Jonas T Neary M Giridharan GA. Cavopulmonary assist: Long-term reversal of the Fontan paradox. J Thorac Cardiovasc Surg. (2019) 158:1627–36. 10.1016/j.jtcvs.2019.06.112 31564543

[66] Rodefeld MD Coats B Fisher T Giridharan GA Chen J Brown JW. Cavopulmonary assist for the univentricular Fontan circulation: von Karman viscous impeller pump. J Thorac Cardiovasc Surg. (2010) 140:529–36. 10.1016/j.jtcvs.2010.04.037 20561640 PMC2924921

[67] Pekkan K Aka IB Tutsak E Ermek E Balim H Lazoglu I. In vitro validation of a self-driving aortic-turbine venous-assist device for Fontan patients. J Thorac Cardiovasc Surg. (2018) 156:292–301 e7. 10.1016/j.jtcvs.2018.02.088 29666009 PMC6021195

[68] Lacour-Gayet FG Lanning CJ Stoica S Wang R Rech BA Goldberg S. An artificial right ventricle for failing fontan: in vitro and computational study. Ann Thorac Surg. (2009) 88:170–6. 10.1016/j.athoracsur.2009.03.091 19559219

[69] Granegger M Thamsen B Hubmann EJ Choi Y Beck D Valsangiacomo Buechel E. A long-term mechanical cavopulmonary support device for patients with Fontan circulation. Med Eng Phys. (2019) 70:9–18. 10.1016/j.medengphy.2019.06.017 31266678

[70] Cysyk J Clark JB Newswanger R Jhun CS Izer J Finicle H. Chronic in vivo test of a right heart replacement blood pump for failed fontan circulation. ASAIO J. (2019) 65:593–600. 10.1097/MAT.0000000000000888 30299303

[71] Langer R Vacanti JP. Tissue engineering. Science. (1993) 260:920–6. 10.1126/science.8493529 8493529

[72] Miao Y Tian L Martin M Paige SL Galdos FX Li J. Intrinsic endocardial defects contribute to hypoplastic left heart syndrome. Cell Stem Cell. (2020) 27:574–89 e8. 10.1016/j.stem.2020.07.015 32810435 PMC7541479

[73] Hrstka SC Li X Nelson TJ Wanek Program Genetics Pipeline G. NOTCH1-dependent nitric oxide signaling deficiency in hypoplastic left heart syndrome revealed through patient-specific phenotypes detected in bioengineered cardiogenesis. Stem Cells. (2017) 35:1106–19. 10.1002/stem.2582 28142228

[74] Kobayashi J Yoshida M Tarui S Hirata M Nagai Y Kasahara S. Directed differentiation of patient-specific induced pluripotent stem cells identifies the transcriptional repression and epigenetic modification of NKX2-5, HAND1, and NOTCH1 in hypoplastic left heart syndrome. PLoS One. (2014) 9:e102796. 10.1371/journal.pone.0102796 25050861 PMC4106834

[75] Porrello ER Mahmoud AI Simpson E Hill JA Richardson JA Olson EN. Transient regenerative potential of the neonatal mouse heart. Science. (2011) 331:1078–80. 10.1126/science.1200708 21350179 PMC3099478

[76] Bergmann O Bhardwaj RD Bernard S Zdunek S Barnabe-Heider F Walsh S. Evidence for cardiomyocyte renewal in humans. Science. (2009) 324:98–102. 10.1126/science.1164680 19342590 PMC2991140

[77] Ishigami S Ohtsuki S Tarui S Ousaka D Eitoku T Kondo M. Intracoronary autologous cardiac progenitor cell transfer in patients with hypoplastic left heart syndrome: the TICAP prospective phase 1 controlled trial. Circ Res. (2015) 116:653–64. 10.1161/CIRCRESAHA.116.304671 25403163

[78] Tarui S Ishigami S Ousaka D Kasahara S Ohtsuki S Sano S. Transcoronary infusion of cardiac progenitor cells in hypoplastic left heart syndrome: three-year follow-up of the transcoronary infusion of cardiac progenitor cells in patients with single-ventricle physiology (TICAP) trial. J Thorac Cardiovasc Surg. (2015) 150:1198-207:208 e1–2. 10.1016/j.jtcvs.2015.06.076 26232942

[79] Ishigami S Ohtsuki S Eitoku T Ousaka D Kondo M Kurita Y. Intracoronary cardiac progenitor cells in single ventricle physiology: the perseus (cardiac progenitor cell infusion to treat univentricular heart disease) randomized phase 2 trial. Circ Res. (2017) 120:1162–73. 10.1161/CIRCRESAHA.116.310253 28052915

[80] Parekkadan B Milwid JM. Mesenchymal stem cells as therapeutics. Annu Rev Biomed Eng. (2010) 12:87–117. 10.1146/annurev-bioeng-070909-105309 20415588 PMC3759519

[81] Burkhart HM Qureshi MY Peral SC O'Leary PW Olson TM Cetta F. Regenerative therapy for hypoplastic left heart syndrome: first report of intraoperative intramyocardial injection of autologous umbilical-cord blood-derived cells. J Thorac Cardiovasc Surg. (2015) 149:e35–7. 10.1016/j.jtcvs.2014.10.093 25466856

[82] Burkhart HM Qureshi MY Rossano JW Cantero Peral S O'Leary PW Hathcock M. Autologous stem cell therapy for hypoplastic left heart syndrome: safety and feasibility of intraoperative intramyocardial injections. J Thorac Cardiovasc Surg. (2019) 158:1614–23. 10.1016/j.jtcvs.2019.06.001 31345560

[83] Kaushal S Wehman B Pietris N Naughton C Bentzen SM Bigham G. Study design and rationale for ELPIS: a phase I/IIb randomized pilot study of allogeneic human mesenchymal stem cell injection in patients with hypoplastic left heart syndrome. Am Heart J. (2017) 192:48–56. 10.1016/j.ahj.2017.06.009 28938963

[84] Shin'oka T Matsumura G Hibino N Naito Y Watanabe M Konuma T. Midterm clinical result of tissue-engineered vascular autografts seeded with autologous bone marrow cells. J Thorac Cardiovasc Surg. (2005) 129:1330–8. 10.1016/j.jtcvs.2004.12.047 15942574

[85] Hibino N McGillicuddy E Matsumura G Ichihara Y Naito Y Breuer C. Late-term results of tissue-engineered vascular grafts in humans. J Thorac Cardiovasc Surg. (2010) 139:431-6:6 e1–2. 10.1016/j.jtcvs.2009.09.057 20106404

[86] Drews JD Miyachi H Shinoka T. Tissue-engineered vascular grafts for congenital cardiac disease: Clinical experience and current status. Trends Cardiovasc Med. (2017) 27:521–31. 10.1016/j.tcm.2017.06.013 28754230 PMC5634923

[87] Lee YU Mahler N Best CA Tara S Sugiura T Lee AY. Rational design of an improved tissue-engineered vascular graft: determining the optimal cell dose and incubation time. Regen Med. (2016) 11:159–67. 10.2217/rme.15.85 26925512 PMC4817496

[88] Best C Strouse R Hor K Pepper V Tipton A Kelly J. Toward a patient-specific tissue engineered vascular graft. J Tissue Eng. (2018) 9:2041731418764709. 10.1177/2041731418764709 29568478 PMC5858675

[89] Shimizu T Yamato M Isoi Y Akutsu T Setomaru T Abe K. Fabrication of pulsatile cardiac tissue grafts using a novel 3-dimensional cell sheet manipulation technique and temperature-responsive cell culture surfaces. Circ Res. (2002) 90:e40. 10.1161/hh0302.105722 11861428

[90] Seta H Matsuura K Sekine H Yamazaki K Shimizu T. Tubular cardiac tissues derived from human induced pluripotent stem cells generate pulse pressure in vivo. Sci Rep. (2017) 7:45499. 10.1038/srep45499 28358136 PMC5371992

[91] Miyagawa S Domae K Yoshikawa Y Fukushima S Nakamura T Saito A. Phase I clinical trial of autologous stem cell-sheet transplantation therapy for treating cardiomyopathy. J Am Heart Assoc. (2017) 6(4). 10.1161/JAHA.116.003918 28381469 PMC5532985

[92] Tsuruyama S Matsuura K Sakaguchi K Shimizu T. Pulsatile tubular cardiac tissues fabricated by wrapping human iPS cells-derived cardiomyocyte sheets. Regen Ther. (2019) 11:297–305. 10.1016/j.reth.2019.09.001 31667209 PMC6813561

[93] Biermann D Eder A Arndt F Seoudy H Reichenspurner H Mir T. Towards a tissue-engineered contractile fontan-conduit: the fate of cardiac myocytes in the subpulmonary circulation. PLoS ONE. (2016) 11:e0166963. 10.1371/journal.pone.0166963 27875570 PMC5119816

[94] Park J Anderson CW Sewanan LR Kural MH Huang Y Luo J. Modular design of a tissue engineered pulsatile conduit using human induced pluripotent stem cell-derived cardiomyocytes. Acta Biomater. (2020) 102:220–30. 10.1016/j.actbio.2019.10.019 31634626 PMC7227659

[95] Qureshi MY Cabalka AK Khan SP Hagler DJ Haile DT Cannon BC. Cell-based therapy for myocardial dysfunction after fontan operation in hypoplastic left heart syndrome. Mayo Clin Proc Innov Qual Outcomes. (2017) 1:185–91. 10.1016/j.mayocpiqo.2017.07.002 30225415 PMC6134900

[96] Rupp S Zeiher AM Dimmeler S Tonn T Bauer J Jux C. A regenerative strategy for heart failure in hypoplastic left heart syndrome: intracoronary administration of autologous bone marrow-derived progenitor cells. J Heart Lung Transplant. (2010) 29:574–7. 10.1016/j.healun.2009.10.006 20044280

[97] Nasseri BA Ebell W Dandel M Kukucka M Gebker R Doltra A. Autologous CD133+ bone marrow cells and bypass grafting for regeneration of ischaemic myocardium: the Cardio133 trial. Eur Heart J. (2014) 35:1263–74. 10.1093/eurheartj/ehu007 24497345

[98] Menasche P Vanneaux V Hagege A Bel A Cholley B Parouchev A. Transplantation of human embryonic stem cell-derived cardiovascular progenitors for severe ischemic left ventricular dysfunction. J Am Coll Cardiol. (2018) 71:429–38. 10.1016/j.jacc.2017.11.047 29389360

[99] Yamamoto R Miyagawa S Toda K Kainuma S Yoshioka D Yoshikawa Y. Long-term outcome of ischemic cardiomyopathy after autologous myoblast cell-sheet implantation. Ann Thorac Surg. (2019) 108:e303–e6. 10.1016/j.athoracsur.2019.03.028 30980822

[100] Sung PH Lee FY Tong MS Chiang JY Pei SN Ma MC. The five-year clinical and angiographic follow-up outcomes of intracoronary transfusion of circulation-derived cd34+ cells for patients with end-stage diffuse coronary artery disease unsuitable for coronary intervention-phase i clinical trial. Crit Care Med. (2018) 46:e411–e8. 10.1097/CCM.0000000000003051 29465434

[101] Mann DL Lee RJ Coats AJ Neagoe G Dragomir D Pusineri E. One-year follow-up results from AUGMENT-HF: a multicentre randomized controlled clinical trial of the efficacy of left ventricular augmentation with Algisyl in the treatment of heart failure. Eur J Heart Fail. (2016) 18:314–25. 10.1002/ejhf.449 26555602

[102] Lee LC Wall ST Klepach D Ge L Zhang Z Lee RJ. Algisyl-LVR with coronary artery bypass grafting reduces left ventricular wall stress and improves function in the failing human heart. Int J Cardiol. (2013) 168:2022–8. 10.1016/j.ijcard.2013.01.003 23394895 PMC3748222

[103] Frey N Linke A Suselbeck T Muller-Ehmsen J Vermeersch P Schoors D. Intracoronary delivery of injectable bioabsorbable scaffold (IK-5001) to treat left ventricular remodeling after ST-elevation myocardial infarction: a first-in-man study. Circ Cardiovasc Interv. (2014) 7:806–12. 10.1161/CIRCINTERVENTIONS.114.001478 25351198

[104] Leor J Tuvia S Guetta V Manczur F Castel D Willenz U. Intracoronary injection of in situ forming alginate hydrogel reverses left ventricular remodeling after myocardial infarction in Swine. J Am Coll Cardiol. (2009) 54:1014–23. 10.1016/j.jacc.2009.06.010 19729119

[105] Chung ES Miller L Patel AN Anderson RD Mendelsohn FO Traverse J. Changes in ventricular remodelling and clinical status during the year following a single administration of stromal cell-derived factor-1 non-viral gene therapy in chronic ischaemic heart failure patients: the STOP-HF randomized Phase II trial. Eur Heart J. (2015) 36:2228–38. 10.1093/eurheartj/ehv254 26056125 PMC4554960

[106] Costa MA Pencina M Nikolic S Engels T Templin B Abraham WT. The PARACHUTE IV trial design and rationale: percutaneous ventricular restoration using the parachute device in patients with ischemic heart failure and dilated left ventricles. Am Heart J. (2013) 165:531–6. 10.1016/j.ahj.2012.12.022 23537969

[107] Traverse JH Henry TD Dib N Patel AN Pepine C Schaer GL. First-in-man study of a cardiac extracellular matrix hydrogel in early and late myocardial infarction patients. JACC Basic Transl Sci. (2019) 4:659–69. 10.1016/j.jacbts.2019.07.012 31709316 PMC6834965

[108] Bejleri D Streeter BW Nachlas ALY Brown ME Gaetani R Christman KL. A bioprinted cardiac patch composed of cardiac-specific extracellular matrix and progenitor cells for heart repair. Adv Healthc Mater. (2018) 7:e1800672. 10.1002/adhm.201800672 30379414 PMC6521871

[109] Khetan S Burdick JA. Patterning network structure to spatially control cellular remodeling and stem cell fate within 3-dimensional hydrogels. Biomaterials. (2010) 31:8228–34. 10.1016/j.biomaterials.2010.07.035 20674004

